# Effectiveness of mHealth-Based Nutritional Interventions on Iron Status of Pregnant Women: Systematic Review of Randomized Controlled Trials

**DOI:** 10.2196/81001

**Published:** 2026-04-09

**Authors:** Saba Abraham Belay, Afework Mulugeta Bezabih, Wim Van Petegem, Christophe Matthys

**Affiliations:** 1 Department of Chronic Diseases and Metabolism, Clinical and Experimental Endocrinology Unit, KU Leuven, Herestraat 49 ON1 Bus 902, Leuven, 3000, Belgium, 32 476 72 61 77; 2 Continuing Professional Development Center, College of Health Sciences, Mekelle University, Mekelle, Ethiopia; 3 Department of Nutrition and Dietetics, School of Public Health, College of Health Sciences, Mekelle University, Mekelle, Ethiopia; 4 Engineering Technology Education Research (ETHER) Unit, Group T Leuven Campus, Faculty of Engineering Technology, KU Leuven, Leuven, Belgium; 5 Department of Endocrinology, University Hospitals Leuven, KU Leuven, Leuven, Belgium; 6 Division of Human Nutrition, Faculty of Medicine and Health Sciences, Stellenbosch University, Cape Town, South Africa

**Keywords:** mHealth, mobile health, anemia, pregnancy, systematic review, randomized controlled trials

## Abstract

**Background:**

Anemia is a global health concern. It is disproportionately prevalent among pregnant women in low-resource regions, where iron deficiency is the leading cause. Given the multifactorial nature of anemia, a range of nutritional interventions is recommended. However, effective implementation is often hindered by limited health care access, poor adherence to supplementation, and gaps in nutrition knowledge and counseling. To address these challenges and optimize hemoglobin (Hb) levels among pregnant women, mobile health (mHealth)−based nutritional interventions offer a promising alternative.

**Objective:**

The aim of the study is to review available evidence on the effectiveness of mHealth-based nutritional interventions on iron status (Hb and/or serum ferritin concentration) among pregnant women.

**Methods:**

Searches were conducted in Embase, CINAHL, Cochrane Library, PubMed, Web of Science, and Scopus, and supplemented by snowballing to identify additional relevant studies from citation lists. The key search strings comprised 4 concepts: “mobile health,” “nutritional intervention,” “Hb, anemia or iron deficiency anemia,” and “pregnant women.” Predefined inclusion and exclusion criteria were applied during screening. The methodological quality of included studies was assessed using the Risk of Bias 2 tool. The primary end point was the change in mean Hb concentration or serum ferritin level. Effect sizes (ESs) were calculated as standardized mean differences, including Cohen *d* and Hedges *g*.

**Results:**

Of the 14,284 studies identified, only 11 randomized controlled trials were included. These studies used various modes of delivery, including mobile phone calls (n=1), SMS text messaging (n=3), and mobile apps (n=4), with some using more than 2 modes (n=3). The effect of mHealth-based nutritional interventions on iron status varied significantly. In total, 4 studies demonstrated a large ES (>0.8), with 3 relying on WhatsApp Messenger as an mHealth delivery mode. Approximately 82% (9/11) of the included studies reported a positive effect (*P* values ranging from <.001 to .047) of the intervention on Hb level, whereas 2 studies reported no statistically significant association (*P*=.33 and *P*=.35, respectively). Notably, interventions with the largest ES achieved clinically significant improvements in Hb concentration, with within- and between-group differences exceeding 1 g/dL. However, including behavioral change theories and nutrition-sensitive components was not consistently associated with larger ESs. Due to high heterogeneity (*I*^2^>95%), attributed to variations in mHealth delivery modes, functions, and interactive features across the included studies, meta-analysis could not be performed.

**Conclusions:**

This review demonstrates that mHealth-supported nutritional interventions effectively optimize Hb concentration in pregnant women. While SMS text messaging was less effective in improving Hb concentration, combining it with another mHealth delivery mode, such as phone calls, improved intervention effectiveness. However, the variability in mHealth delivery modes, functions, and interactive features underscores the need for tailored strategies that account for context-specific challenges, digital literacy, and access to technology to enhance effectiveness.

## Introduction

Anemia during pregnancy, defined by the World Health Organization as a hemoglobin (Hb) concentration below 11 g/dL, remains a critical public health problem [1,2]. Globally, anemia affects nearly 36.8% of pregnant women, with the prevalence disproportionately higher in low-resource settings [2-4]. The etiology of anemia is multifactorial, involving nutritional deficiencies, mainly caused by deficiencies in hematopoietic nutrients such as vitamins A, B_2_, B_6_, B_12_, C, D, E, and folate, as well as minerals like iron, copper, and zinc; infections; chronic disease; inflammation; and genetic Hb disorders [5-7]. Iron deficiency anemia alone accounts for over half of cases, driven by increased gestational iron demands, poor dietary intake, and dietary habits such as inadequate consumption of heme iron sources (animal-based foods) and high intake of iron inhibitors, including polyphenols (tea and coffee) and phytates (whole grains) [4-7].

Maternal anemia is associated with adverse perinatal outcomes, including stillbirth, preterm birth, low birth weight, intrauterine growth retardation, and high neonatal mortality rates [8-10]. Furthermore, anemia during pregnancy is linked to neurodevelopmental disorders, attention-deficit/hyperactivity disorder, and intellectual disability in children [11]. In mothers, anemia raises the risk of pre-eclampsia, postpartum depression, and maternal mortality [3].

Recognizing the consequences of anemia, countries have been implementing both nutrition-specific and nutrition-sensitive solutions aimed at preventing and managing the condition. The nutrition-specific interventions focus on the most immediate causes of anemia, particularly inadequate intake of iron and other hematopoietic nutrients. Strategies include adhering to iron and folic acid supplementation, promoting dietary diversity, implementing iron fortification programs, and providing nutritional education and counseling. Conversely, nutrition-sensitive interventions tackle the underlying causes of anemia by addressing food security, ensuring access to adequate health services, and maintaining a safe and hygienic environment [5,12,13].

The effectiveness of iron and folic acid supplementation and dietary intake is closely associated with the dietary behavior of individuals. According to Marijn Stok et al [14], dietary behavior relates to all phenomena related to food choices, eating habits, and dietary intake patterns. Various studies have highlighted the pivotal role of nutritional education and counseling in influencing dietary behaviors, enhancing nutritional knowledge and attitudes, and promoting anemia prevention and management strategies [3,15,16]. A meta-analysis focused on the impact of nutritional education and counseling on iron status of pregnant women in low- and middle-income countries (LMICs) found that women who received nutritional education and counseling experienced an average increase of 0.88 g/dL (95% CI 0.63-1.13 g/dL; *P*<.001) in Hb concentration and a 34% lower risk of anemia compared to the control group [16].

Despite the potential of nutritional education and counseling in improving health and dietary behavior, a review targeting 4 countries (2 in Africa and 2 in Asia) found that pregnant women were not reached frequently enough, were not counseled adequately, and consequently lacked motivation, nutritional knowledge, and optimal dietary practice [17]. These gaps highlight the need for nutritional education and counseling to be supported by innovative solutions, such as mobile health (mHealth), to effectively influence dietary behavior through improving knowledge, attitudes, and dietary practices [5,18].

mHealth, defined as the use of mobile technologies in health care, has emerged as a promising tool for improving the accessibility, affordability, and effectiveness of nutrition care services in both high-income countries and LMICs [18,19]. Furthermore, nowadays, the delivery of health behavior change interventions increasingly relies on mHealth technologies due to their widespread penetration across socioeconomic levels [20]. For example, an umbrella review of 47 meta-analyses, comprising 507 randomized controlled trials (RCTs), documented an impact of mHealth on dietary behavior. According to this review, mHealth interventions have small-to-moderate improvements across various dietary outcomes [21].

However, while several reviews have explored the role of mHealth interventions in improving maternal and child health outcomes, there remains a lack of systematic reviews specifically focusing on mHealth-based nutritional interventions and their impact on the iron status of pregnant women. To our knowledge, no review has identified, appraised, and synthesized the existing evidence focused on nutritional interventions supported by mHealth in optimizing the iron status of pregnant women.

Therefore, this systematic review aims to evaluate the effectiveness of mHealth-based nutritional interventions on the iron status of pregnant women through a synthesis of evidence from existing RCTs. Additionally, this review examined the effects of mHealth-based interventions on secondary outcomes, including adherence to iron and folic acid supplementation, dietary intake, gestational weight gain, and nutritional knowledge, attitudes, and practices, as well as patient-centered implementation outcomes reported in the included studies. The findings are intended to inform and guide the development of future mHealth interventions with relevant evidence to address anemia during pregnancy.

## Methods

### Overview

The review protocol was registered in PROSPERO (International Prospective Register of Systematic Reviews; CRD42025627769) on January 17, 2025, with prespecified primary and additional secondary outcomes (adherence to iron and folic acid supplementation, dietary intake, gestational weight gain, and nutritional knowledge and practice). Attitudes and implementation outcomes were introduced later as additional secondary outcomes not included in the original registration. The paper was structured following the updated guidelines for PRISMA (Preferred Reporting Items for Systematic Reviews and Meta-Analyses; Checklist 1) statement and methodological considerations of conducting a systematic review of RCTs [22].

### Search Strategy

The searches were conducted in CINAHL (via EBSCOhost), Embase, PubMed (including MEDLINE, via NCBI), CENTRAL (via Cochrane Library), Scopus, and Web of Science Core Collection using a predeveloped search strategy (Multimedia Appendix 1). A combination of indexed terms, database-specific keywords, and MeSH terms was used to improve the search. The search query retrieved all studies that included key terms such as mHealth, nutritional interventions, Hb, anemia or iron deficiency anemia, and pregnant women in either the title, abstract, or keywords. Besides, references and citation lists of all included studies and relevant systematic reviews that met our eligibility criteria were further screened through snowballing.

### Eligibility Criteria

The population, intervention, comparison, and outcome scheme was used in this review to formulate the review question and define the eligibility criteria. In addition, the study design, setting, publication language, and period were prespecified as part of the inclusion and exclusion criteria (Multimedia Appendix 2). We included individual and cluster randomized controlled trials (cRCTs), as these study designs are considered the gold standard for clinical research, generating robust and reliable conclusions. This is due to the random allocation of participants to competing interventions and the analytic approaches that support causal inference [23,24]. In contrast, we excluded nonrandomized studies of interventions, including quasi-RCTs, non-RCTs, cohort studies, and case-control studies, as these designs present methodological challenges. Effect estimates obtained from nonrandomized studies of interventions are more subject to additional sources of bias, such as confounding [23,25].

Trials fulfilling the inclusion criteria and published between January 1, 2003, and February 28, 2025 (the date of the final search) were included. All pregnant women were included, regardless of gestational age, Hb level, age, or country of residence. We included studies reporting mean Hb concentration as part of their study outcomes. Thus, we described iron status in terms of mean Hb concentration (expressed in g/dL) or iron deficiency anemia, defined as Hb <11 g/dL or serum ferritin <12 μg/L) [1].

Nutritional interventions delivered either as standalone interventions (using only mHealth components) or as part of a comprehensive intervention package (mHealth combined with other non-mHealth components) were included in the review. We considered various mobile technology tools that facilitate communication in remote areas, including phone calls, video calls, SMS text messages, multimedia messaging services, mobile apps, and social media platforms (eg, WhatsApp, Facebook, Telegram, and TikTok). We also included studies comparing mHealth-based nutritional interventions with standard antenatal care, described as “usual care,” “standard care,” “routine care,” or “standard antenatal care.”

Non–peer-reviewed papers were excluded due to concerns about scientific consistency, methodological compliance, and reliability, which are better ensured in peer-reviewed publications. Nutrition interventions that were supported or delivered by any means other than mHealth technologies were also excluded. Additionally, we excluded studies published before 2003, as the widespread adoption of mHealth-based interventions and the emergence of related publications occurred after this period [26]. This criterion ensures that included studies reflect contemporary mHealth interventions using mobile technologies.

### Study Selection and Data Extraction

The retrieved records were imported into EndNote (version 21; Clarivate) software for reference management and duplicate removal. After duplicates were manually verified and removed by 1 author (SAB), the remaining papers were transferred to Rayyan (Qatar Computing Research Institute) for systematic screening.

Two authors (SAB and CM) independently screened the titles and abstracts and categorized papers as relevant (met the eligibility criteria), irrelevant (did not meet the eligibility criteria), and uncertain (inconclusive information on eligibility criteria) following the removal of duplicates. Both authors then reviewed and assessed “potentially relevant” and “uncertain” papers in full text based on the eligibility criteria. Any discrepancies arising during the selection process were resolved through discussion and, when necessary, the involvement of a third reviewer (AMB).

Based on the guidelines in the Cochrane Handbook for Systematic Reviews of Interventions, a comprehensive data extraction form was developed by 1 author (SAB) and subsequently reviewed and refined by 3 authors (CM, WVP, and AMB). The form was piloted on at least 2 included studies to ensure reliability and reproducibility.

The following information was extracted from the included studies:

- Study identifiers: author details and publication details.
- Participant characteristics: age (in years), gestational age (in weeks), and anemia status (Hb concentration in g/dL).
- Study methodology: study setting, sample size and sampling procedures, study design, eligibility criteria, processes for randomization, allocation and blinding, and statistical analysis details.
- Intervention description: characteristics and components of the intervention, intervention delivery methods, duration of follow-up, and details of the comparison intervention.
- Intervention outcomes: attrition rate, continuous data (mean and SD) of Hb concentration, and any reported secondary outcomes.

Following the development of the extraction form, data were extracted by 1 author (SAB) and independently verified by 2 authors (CM and AMB). Discrepancies were resolved through consensus or, if necessary, by consulting a fourth reviewer (WVP). All relevant information was obtained from the full texts of the included studies. Corresponding authors were contacted when clarification was required or when data were missing.

### Evaluation of the Methodological Quality of the Studies

Two authors (SAB and CM) independently assessed the methodological quality of the included studies using the revised Risk of Bias 2 tool [27]. In accordance with Risk of Bias 2 guidance, the risk of bias was assessed at the outcome level rather than the study level. Specifically, we evaluated the risk of bias for the results related to the primary outcome of interest.

The tool uses five domains to incorporate all types of bias currently considered to affect the results of RCTs. The domains include (1) risk of bias arising from the randomization process, (2) risk of bias due to deviations from the intended interventions, (3) risk of bias due to missing outcome data, (4) risk of bias in measurement of the outcome, and (5) risk of bias in selection of the reported result, followed by the assessment of the overall risk of bias. On this basis, studies were categorized as having a “high” risk of bias (high risk in at least 1 domain or some concerns across multiple domains), “some concerns” (some concerns in at least 1 domain without any domain rated as high risk), or a “low” risk of bias (low risk across all domains). Any disagreements during the quality assessment were resolved through discussion, involving third and fourth reviewers (AMB and WVP) as needed. Additionally, an independent reviewer (Kidu Gidey) cross-checked the risk-of-bias judgments within domains.

### Dealing With Missing Data

For missing data in the included studies (eg, SDs or means), we first attempted to contact the corresponding authors via email to obtain the required information. If data could not be retrieved, we followed guidance from the Cochrane Handbook to derive missing statistics from other reported measures using the generic inverse variance method [23]. For instance, missing SDs were calculated from CIs for means, SEs, *t* statistics, or *P* values when available. In addition, when data were symmetrically distributed, reported medians were considered as a reasonable substitute for means. Studies with missing data were not directly excluded; instead, they were considered in sensitivity analyses to assess the robustness of the findings. We excluded studies with missing data that were irretrievable and judged to be at high risk of bias from effect size (ES) estimation.

### Data Synthesis

The characteristics of mHealth interventions are described in terms of mHealth function, interaction feature, and delivery mode.

#### mHealth Function

We categorized mHealth functions in each study following the classification proposed by Knop et al [28], which outlines 12 mHealth functions. Among these, we focused on the “client education and behavior change communication” function [28]. To systematically analyze the behavior change components within this function, we mapped each intervention to the corresponding behavior change techniques (BCTs) derived from the BCT Taxonomy v1, which includes 93 hierarchically clustered techniques [29]. This approach enables integration of the mHealth function classification with specific and theory-based BCTs.

#### mHealth Interaction Features

In addition to describing the mHealth feature, it is crucial to examine the level of interaction between the patient or participants and these features. As reported by Knop et al [28], interactions are categorized as unidirectional (1-way communication), bidirectional (2-way communication), and multidirectional (complex interactions involving multiple stakeholders). Following the description by Donevant et al [30]*,* which aligns with existing terminology, we described these interactions as passive and interactive features. The passive feature does not require any additional response or action from the patient within the mHealth app, including 1-way text messaging and reminders. In contrast, interactive features enable patients to respond or engage on the matter, including interactive prompts and 2-way communication via texting, email, phone calls, and other mobile apps [30].

#### Measures of Mean Hb Concentration (g/dL)

The primary end point of this review was the change in mean Hb concentration (g/dL). For each included study, we calculated the mean change in Hb concentration (g/dL) from baseline. We assessed within-group differences (pre- vs postintervention) and between-group differences based on postintervention mean Hb concentrations (intervention vs control).

#### Measures of Statistical Significance (*P* Value)

For the evaluation of effectiveness, an intervention was classified as effective if the mHealth-based nutritional intervention resulted in statistically significant (*P*<.05) outcomes, as compared to the control group. The intervention was classified as ineffective if there was no statistical difference between the intervention and control groups.

#### Measures of Intervention Effect

The standardized mean difference, a commonly used ES measure in RCTs, including 6 cRCTs and 5 individual RCTs, was calculated. For consistency across study designs, we estimated the standardized mean difference using both Cohen *d* and Hedges *g*. Cohen *d* is a standard metric for ES estimation, while Hedges *g* is similar but incorporates a correction factor to reduce bias in small sample sizes [31,32].

The ES (Cohen *d*) for continuous outcomes was calculated using the formula: $d=\frac{\left(M1-M2\right)}{SDp}$, where M₁ and M₂ represent the mean Hb concentrations of the intervention and control groups, respectively, and SD_p_ is the pooled SD of the 2 groups [31]. Group-level summary statistics, which had already accounted for the study design in the original analysis, were used to estimate the ES in cRCTs. Consequently, no additional clustering adjustments were made.

We summarized the intervention’s effect on iron status separately for each study. The magnitude of Hedges *g* is interpreted using Cohen convention, where an ES of <0.20 is small, 0.50 to 0.80 is medium, and scores >0.80 are large [31]. Comprehensive Meta-Analysis software was used to calculate the ES.

#### Heterogeneity

Heterogeneity was quantified using the chi-square and the *I*^2^ statistic. These measures assess the variability in the intervention effects across studies. Following Cochrane guidelines, a chi-square greater than the *df* and a small *P* value (eg, *P*<.05) indicate evidence of heterogeneity of intervention effects (variation in effect estimates beyond chance) [23]. *I*^2^ values were interpreted as: 0% to 40%: might not be important, 30% to 60%: moderate heterogeneity, 50% to 90%: substantial heterogeneity, and 75% to 100%: considerable heterogeneity [23]. Quantitative synthesis was considered only when heterogeneity was low to moderate. With substantial or considerable heterogeneity, results were synthesized narratively.

### Secondary Outcome

Based on a review of the existing literature, we defined the following secondary outcomes. Adherence to iron and folic acid supplementation was defined as the consumption of at least 4 iron-folic acid tablets per week for the recommended period [33]. Dietary intake was defined as food and nutrient consumption at an individual, household, or population level over a period [34,35]. Gestational weight gain was characterized as the recommended range of weight a pregnant woman should gain during pregnancy to optimize maternal and child health outcomes [36]. Nutritional knowledge was defined as the knowledge of nutrition, including the ability to recall nutrition and diet-related terminology [37]. Attitude was described as an individual’s feeding or eating behavior influenced by feelings, motivations, perceptions, and thoughts [37]. Practice was operationalized as an individual’s actions that could affect his or her nutrition, such as eating, feeding, cooking, and selecting foods [37].

In addition, the following implementation outcomes were defined based on the framework proposed by Proctor et al [38]. Appropriateness was defined as the perceived relevance or compatibility of the intervention for the target groups to address the issue or problem. Acceptability or satisfaction was defined as the perception among the target groups that a given practice or intervention is agreeable, palatable, or satisfactory. Implementation was defined as the cost impact of an implementation effort. Feasibility was defined as the extent to which the newly developed intervention can be successfully used or carried out within a given setting. Adoption or uptake was defined as the intention, initial decision, or action to try or use the intervention. Fidelity was defined as the degree to which an intervention was implemented as intended by its developers. Sustainability was defined as the extent to which a newly implemented intervention is maintained or institutionalized within a service setting over time.

## Results

### Search Results

**Figure 1.**
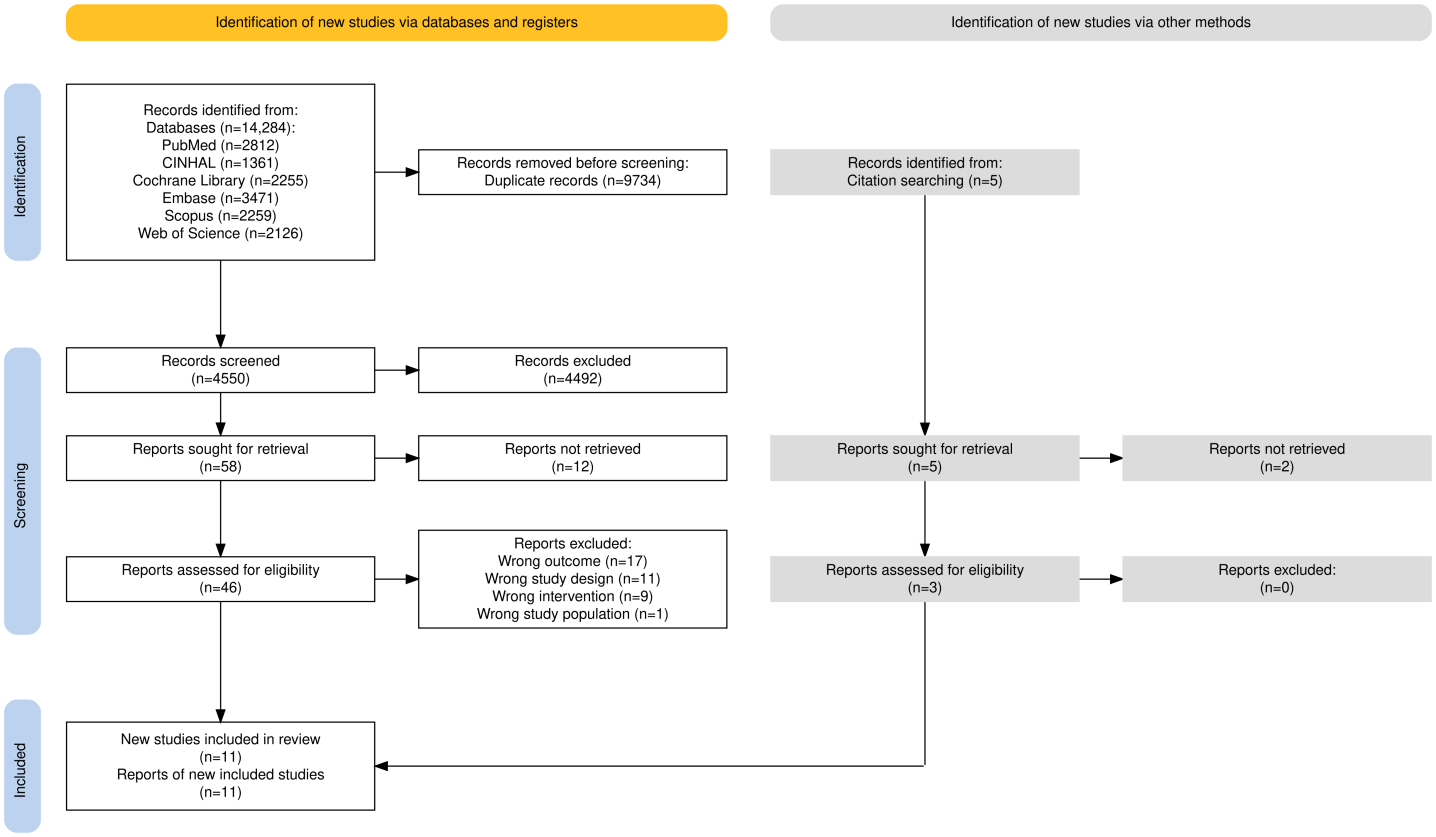
PRISMA (Preferred Reporting Items for Systematic Reviews and Meta-Analyses) flow diagram of study selection process.

As shown in Figure 1 [39], a total of 14,284 studies were extracted from 6 databases, and 5 additional papers through citation searching. We screened titles and abstracts of 4550 papers. Of these, we assessed the full text of 46 papers against the eligibility criteria. Following the full-text assessment, 11 studies published between 2018 and 2023 were included in this review. A total of 38 records were excluded, with detailed reasons for exclusion provided in the PRISMA diagram.

### Study Characteristics

Table 1 summarizes characteristics across 11 included studies. Among these, 6 studies [40-45] were individual RCTs conducted in health facility settings. Of the 5 cRCT studies, only 1 study [46] was conducted at the community level, while the remaining 4 [15,47-49] were conducted in health facilities. In terms of geographical distribution, the majority (7 studies) [15,41,43-46,48] were conducted in low- and lower-middle-income countries, while the remaining 4 studies [40,42,47,49] were conducted in high- and upper-middle-income countries, according to the World Bank classification [50]. Only 2 continents were represented in this systematic review, with Asia contributing 9 (81.8%) studies, and the remaining 2 (18.2%) studies were conducted in Africa. The sample size in the included studies ranged from 59 in Thailand [42] to 413 participants in Nepal [46], with a total of 2191 (mean 199.2, SD 104.1) study participants.

**Table 1.** The characteristics of included studies.

Study and publication year	Study location	Study design	Sample size	Inclusion criteria			Exclusion criteria
				Age (years)	Gestational weeks	Hb^a^ level (g/dL)	Pregnant women with the following conditions were excluded from the study
Elsharkawy et al (2022) [40]	Saudi Arabia	RCT^b^	n=196 Intervention: 98 Control: 98	18‐45	14‐16	<11	Unable to read or write, had multiple fetuses, hereditary anemia, or chronic illnesses, had a Hb level of <7 g/dL, or had been on iron and folic acid supplementation for more than 1 week.
Sontakke et al (2022) [41]	India	RCT	n=240 Intervention: 120 Control: 120	—^c^	13‐28	8‐11	With hemoglobinopathies or proven worm infestations.
Singh et al (2020) [46]	Nepal	cRCT^d^	n=413 Intervention: 199 Control: 214	15‐45	13‐28	—	—
Abd Rahman et al (2022) [47]	Malaysia	cRCT	n=104 Intervention: 50 Control: 54	20‐40	13‐24	7‐10.9	With underlying chronic diseases, symptoms of anemia, or plans for abortion in the current pregnancy.
Xuto et al (2022) [42]	Thailand	RCT	n=59 Intervention: 30 Control: 29	18‐40	11-13	—	With a twin pregnancy and unwilling to participate due to threatened abortion or termination of pregnancy.
Sharma et al (2022) [44]	India	RCT	n=143 Intervention: 71 Control: 72	—	<13	—	—
Washington et al (2023) [48]	Liberia	cRCT	n=150 Intervention: 75 Control: 75	—	—	—	With chronic illnesses.
Wakwoya et al (2023) [15]	Ethiopia	cRCT	n=326 Intervention:163 Control: 163	—	<16	—	Had chronic illnesses, refused to provide verbal consent, or intended to leave the study area.
Ahmad et al (2022) [49]	Indonesia	cRCT	n=110 Intervention: 55 Control: 55	—	>28	—	—
Sharma et al (2023) [43]	India	RCT	n=250 Intervention: 127 Control: 123	—	14‐24	<11	Had a history of obstetric, medical, or psychological problems.
Abujilban et al (2019) [45]	Jordan	RCT	n=200 Intervention: 100 Control: 100	18‐45	13‐28	<10.5	Illiterate or had a history of obstetric, medical, or psychological problems.

a Hb: hemoglobin.

b RCT: randomized controlled trial.

c No report.

d cRCT: cluster randomized controlled trial.

The age range of participants at baseline was reported in 5 studies [40,42,45-47], ranging from 15 to 45 years. Except for 1 study [48], all studies provided information on the gestation age (in weeks) of the participants. Additionally, 5 studies [40,41,43,45,47] selected the participants based on their Hb levels (Hb <11 g/dL), while the remaining 6 studies did not consider the anemia status of the participants as a selection criterion.

### Description of the mHealth Interventions

Descriptions of the mHealth and nutritional interventions from the reviewed studies are summarized in Tables2  3. Among the included studies, 5 studies [40,43,45,47,49] used WhatsApp Messenger to deliver text, audio, and video-based educational messages to pregnant women in the intervention group. Different modes of delivery, including mobile phone calls, SMS text messaging, and mobile apps, were reported. Some studies used more than 1 mode of delivery [43,48,49]. Approximately 91.9% of the included studies used client education and behavior change communication to convey educational information to the target groups.

**Table 2.** Summary of mobile health (mHealth) characteristics^a^.

Study	Delivery mode	mHealth function	Interaction feature	Intervention (mHealth group) received	Follow-up period (weeks)
Elsharkawy et al [40]	WhatsApp texts	Education Reminder^b^ Feedback	Interactive	One educational message, four medication reminders, and more than three participant feedback	12
Sontakke et al [41]	Phone calls	Reminder Feedback	Interactive	Biweekly phone calls and reminder notifications	12
Singh et al [46]	SMS or texting	Education	Passive	One message every 2 weeks (4‐6 months), weekly thereafter until childbirth	12
Abd Rahman et al [47]	WhatsApp video	Education	Passive	Daily 3‐ to 5-minute educational video (6 days), followed by weekly reminders (weeks 2‐5)	12
Xuto et al [42]	SMS or texting	Education	Passive	Two text messages per week (13‐40 gestational weeks)	28
Sharma et al [44]	Mobile app	Education Reminder Feedback	Passive	Nutritional advice, test tracking, data input, graphical or textual visualization of data, and medication reminder	Until delivery
Washington et al [48]	SMS and phone calls	Education	Passive	Biweekly “antenatal care telereminder” texts and calls	12
Wakwoya et al [15]	SMS or texting	Education	Passive	Weekly serial SMS messages	12
Ahmad et al [49]	Phone calls and WhatsApp text	Education Feedback	Interactive	Educational flyers via WhatsApp and telephonic nutrition education	12
Sharma et al [43]	SMS or texting, phone calls, and WhatsApp audio	Education Reminder Feedback	Interactive	Four SMS, one WhatsApp audio message, and six weekly calls	4
Abujilban et al [45]	WhatsApp video	Education	Passive	15-minute educational video	12

a All participants in the control groups received routine antenatal care.

b Reminder: medication reminder.

**Table 3.** Summary of mobile health (mHealth) components, nutritional interventions, and behavior change techniques (BCTs) or behavior change theory applied.

Study	mHealth component	Other components	Nutrition interventions or topics covered by mHealth		BCT taxonomy applied	Behavior change theory
			Nutrition interventions (sensitive)	Nutrition interventions (specific)		
Elsharkawy et al [40]	Educational texts	PowerPoint slides, guidelines, brochures	IFAS^a^, dietary advice, and anemia prevention and management	—^b^	Knowledge shaping, prompts and cues, feedback and monitoring, social support	—
Sontakke et al [41]	Phone call reminders	—	IFAS	—	Prompts and cues, goal setting, feedback and monitoring, social support	—
Singh et al [46]	Educational texts	HCP^c^ training	General nutrition advice	Health care service use	Knowledge shaping	—
Abd Rahman et al [47]	Educational videos and reminders	—	IFAS, dietary advice, and anemia prevention and management	—	Knowledge shaping, natural consequences	HBM^d^
Xuto et al [42]	Educational texts	—	Dietary advice	—	Knowledge shaping	—
Sharma et al [44]	Apps-based content	—	IFAS, dietary advice, and malaria prevention	Reproductive health care	Knowledge shaping, feedback and monitoring, prompts and cues, social support	—
Washington et al [48]	Educational texts and calls	HCPs or guideline manual	IFAS, dietary advice, malaria prevention, and intestinal parasite control	Hygiene and health care service use	Knowledge shaping, prompts and cues, social support	—
Wakwoya et al [15]	Educational texts	Face-to-face nutritional counseling and brochures	IFAS, dietary advice, use of iodized salt, malaria, and intestinal parasite prevention and control	Hygiene and health care service use	Knowledge shaping	HBM
Ahmad et al [49]	Educational calls and texts	Nutrition booklet and food monitoring card	IFAS and dietary advice	Hygiene	Knowledge shaping, prompts and cues, feedback and monitoring, social support	TPB^e^
Sharma et al [43]	Reminder calls, texts, and audio messages	—	IFAS	—	Feedback and monitoring, prompts and cues	—
Abujilban et al [45]	Educational videos	—	Iron supplementation and dietary management	—	Knowledge shaping	—

a IFAS: iron and folic acid supplementation.

b No report.

c HCP: health care provider.

d HBM: health belief model.

e TPB: theory of planned behavior.

Regarding mHealth interaction features, 7 studies [15,42,44-48] incorporated a passive feature (unidirectional communication approach), using push technology to deliver educational content or reminders to the target groups. In contrast, 5 of the studies [40,41,43,44,49] used an interactive feature (bidirectional communication; Table 2).

The follow-up duration across studies ranged from 4 weeks [43] to 28 weeks [42], with a mean of 12.8 (SD 5.9) weeks estimated from 10 studies. One study [44] did not provide a clear intervention duration. Approximately 82% of studies implemented an intervention follow-up period of 12 weeks.

This review also identified a range of BCTs across the included studies, as presented in Table 3. Approximately 54.5% of the studies [40,41,43,44,48,49] applied 2 or more BCTs, with the most frequently used techniques including shaping knowledge, prompts and cues, and feedback and monitoring. Additionally, only 3 studies [15,47,49] incorporated a behavior change theory, such as the health belief model and the theory of planned behavior.

In total, 5 studies [15,40,46,48,49] supplemented their mHealth interventions with additional strategies such as face-to-face presentations, nutritional counseling, guideline and brochure distribution, and health care provider capacity-building training sessions to enhance intervention effectiveness. All studies incorporated nutrition-specific topics, conveying advice on key areas such as iron and folic acid supplementation, dietary intake, and strategies for preventing and managing anemia, malaria, and intestinal parasites. However, nutrition-sensitive topics related to health care service use, hygiene, and reproductive health were covered in only 5 studies [15,44,46,48,49].

### Risk of Bias in Included Studies

**Figure 2.**
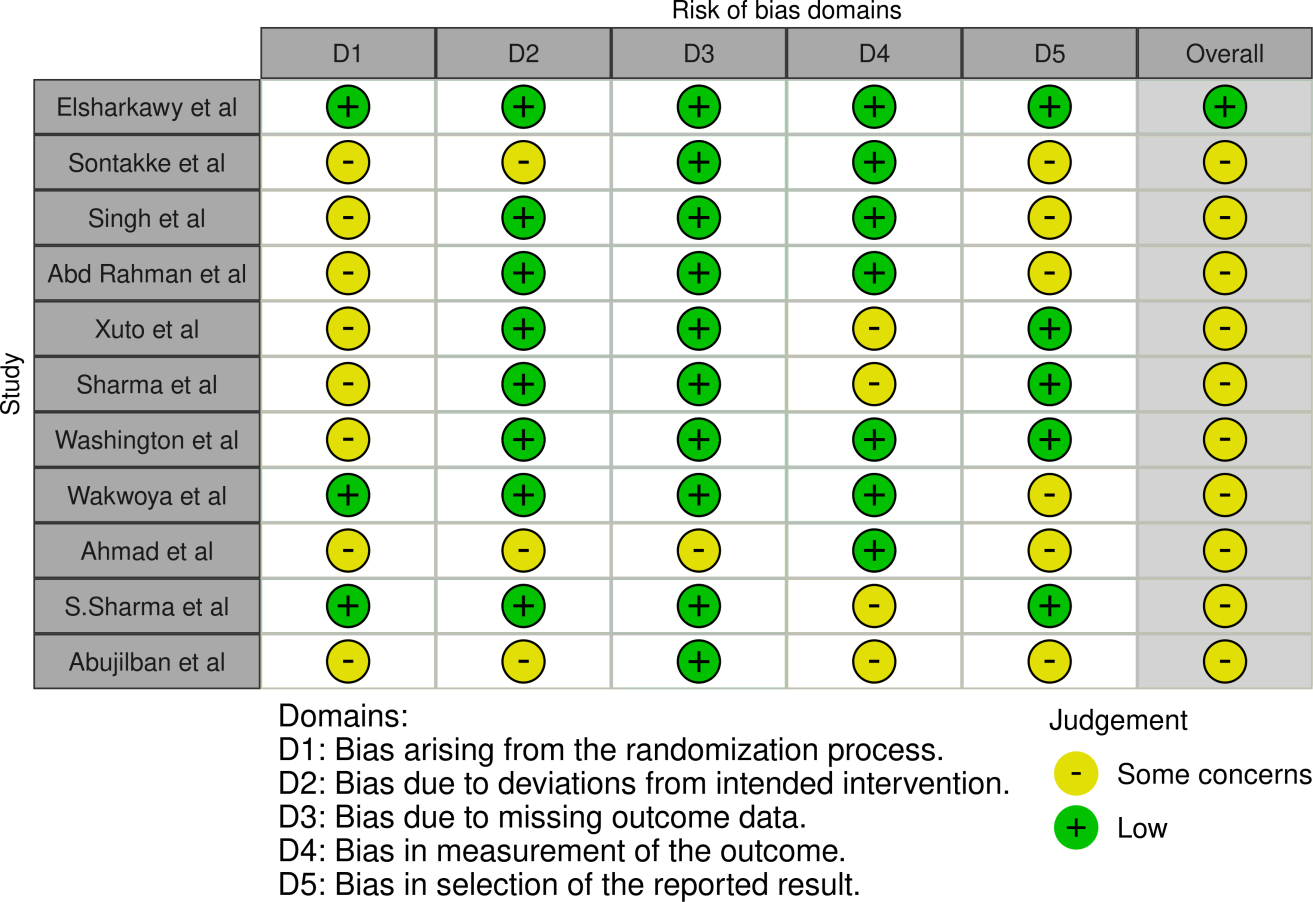
Risk of bias plot for included randomized controlled trials [15,40-49].

The methodological quality of the studies varied noticeably. A total of 90.9% of studies were judged to have some concern regarding the risk of bias, with only 1 study classified as having a low risk of bias. Approximately 8 (72.7%) of the studies did not report the method used for allocation sequence concealment, and more than half (54.5%) demonstrated a bias in the selection of reported results. Blinding practice differed across the included studies. In total, 5 studies [15,40,42,47,48] were able to blind participants. The risk of bias for each included study is described in Figure 2 [51], and detailed descriptions of the risk of bias judgments are provided in Multimedia Appendix 3 [15,40-49].

### Effects of mHealth-Based Nutritional Interventions

**Figure 3.**
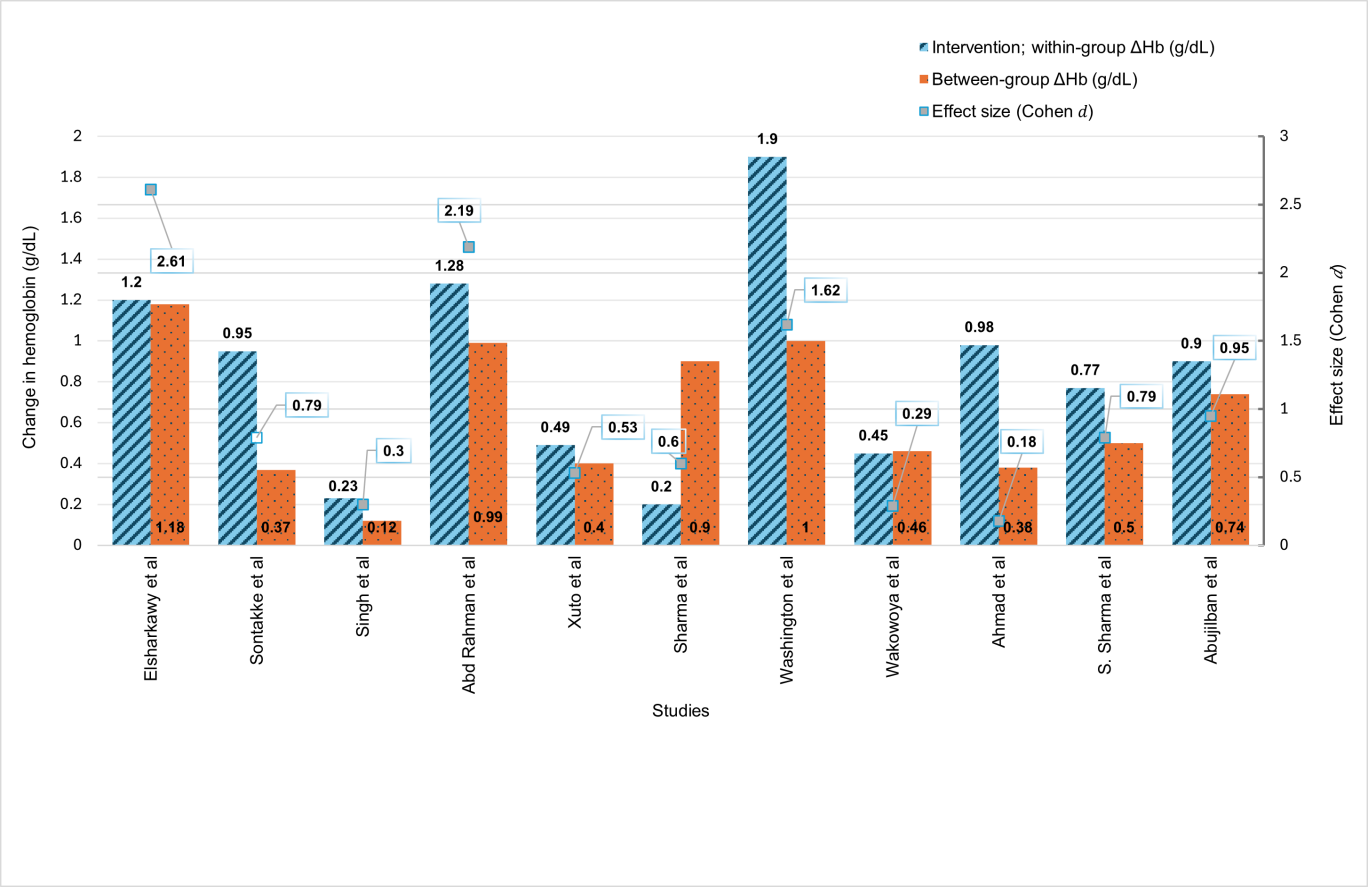
Mean hemoglobin change (∆Hb) within and between groups (g/dL) and corresponding effect sizes (Cohen *d*) across included studies [15,40-49].

Of the 11 included studies, 10 reported participants’ Hb concentration (g/dL) using mean and SD to describe iron status, while 1 study [43] used the median and IQR to report pre- and postintervention changes in Hb concentration. As Figure 3 shows, the highest mean Hb concentration difference within the intervention group (1.9 g/dL) was reported by Washington et al [48], while the smallest difference (0.2 g/dL) was observed in the study of Sharma et al [44]. The smallest between-group difference (intervention vs control) was 0.19 g/dL [49], whereas the largest was 1.18 g/dL [40]. Notably, interventions with the largest ES (eg, ES=2.61, 95% CI 2.23-3.00; ES=2.19, 95% CI 1.70-2.68; ES=1.62, 95% CI 1.25-1.99) achieved clinically meaningful improvements in Hb concentration. These increases exceeded the widely recognized 1 g/dL threshold, indicative of a positive therapeutic response to anemia in pregnant women, and are associated with significant improvements in maternal health outcomes [52,53].

Almost 82% (9/11) of studies showed a positive association between intervention and Hb concentration. However, 2 studies [42,49] with small ES (Cohen *d*=0.5) found no association between the mHealth intervention and the Hb concentration in the intervention group (*P=*.33 and *P*=.35, respectively; Table 4).

**Table 4.** Summary of study effect size for hemoglobin level (g/dL).

Studies	Mode of delivery	Control			Intervention				Effect size^a^	
		Sample size	Preintervention	Postintervention	Sample size	Preintervention	Postintervention		Cohen *d* with 95% CI	Hedges *g*
		n (%)	Mean (SD)	Mean (SD)	n (%)	Mean (SD)	Mean (SD)	*P* value		
Elsharkawy et al [40]	WTM^b^	98 (9)	9.99 (0.32)	10.01 (0.37)	98 (8.8)	9.96 (0.30)	11.16 (0.50)	.001	2.61 (2.23 to 3.00)	2.60
Sontakke et al [41]	MPC^c^	120 (11.1)	9.48 (0.68)	10.06 (0.70)	120 (10.8)	9.74 (0.68)	10.69 (0.89)	.001	0.79 (0.52 to 1.05)	0.78
Singh et al [46]	TM^d^	199 (18.4)	11.00 (1.15)	11.11 (1.10)	214 (19.3)	11.2 (1.08)	11.43 (1.00)	.02	0.30 (0.11 to 0.50)	0.30
Abd Rahman et al [47]	WVM^e^	50 (4.6)	10.12 (0.66)	10.41 (0.44)	54 (4.9)	10.20 (0.51)	11.48 (0.53)	.001	2.19 (1.70 to 2.68)	2.17
Xuto et al [42]	TM	29 (2.7)	11.66 (1.01)	11.75 (1.37)	30 (2.7)	11.95 (0.68)	12.44 (1.25)	.33^f^	0.53 (0.01 to 1.05)	0.52
Sharma et al [44]	Mapp^g^	72 (6.6)	11.60 (1.30)	10.90 (1.40)	71 (6.4)	11.30 (1.50)	11.50 (1.40)	.03	0.43 (0.10 to 0.76)	0.43
Washington et al [48]	TM and PC^h^	75 (6.9)	10.80 (0.92)	11.70 (0.65)	75 (6.8)	10.90 (0.94)	12.80 (0.71)	.047	1.62 (1.25 to 1.99)	1.61
Wakwoya et al [15]	TM	163 (15)	12.20 (1.44)	12.19 (1.16)	163 (14.7)	12.08 (1.15)	12.53 (1.18)	.01	0.29 (0.07 to 0.51)	0.29
Ahmad et al [49]	PC and TM	55 (5.1)	11.35 (1.25)	11.95 (1.16)	55 (5)	11.16 (0.90)	12.14 (0.96)	.35^f^	0.18 (−0.20 to 0.55)	0.18
Sharma et al [43]	TM, PC, and WAM^i^	123 (11.3)	9.93 (0.75)	10.20 (0.60)	127 (11.5)	9.93 (1.05)	10.70 (0.67)	.001	0.79 (0.53 to 1.04)	0.78
Abujilban et al [45]	WVM	100 (9.2)	9.55 (0.86)	9.71 (1.08)	100 (9)	9.66 (0.70)	10.56 (0.96)	.01	0.83 (0.54 to 1.12)	0.83

a Effect size (Cohen *d*): 0-0.1: no effect; 0.2-0.4: small effect; 0.5-0.7: intermediate effect; 0. 8->1: large effect.

b WTM: WhatsApp text message.

c MPC: mobile phone call.

d TM: text message.

e WVM: WhatsApp video message.

f Nonsignificant *P* value (*P*≥.05).

g Mapp: mobile app.

h PC: phone call.

i WAM: WhatsApp audio message.

The ES varied among studies, ranging from ES=2.61 (95% CI 2.23‐3.00) [40] to ES=0.18 (95% CI −0.20 to 0.55) [49]. Among the studies with a small ES (Cohen *d*=0.5), the majority used SMS text messages as their mode of delivery [15,46]. Studies with the highest ESs (ES=2.61, 95% CI 2.23‐3.00 and ES=2.20, 95% CI 1.70-2.68) used WhatsApp as an mHealth delivery mode, incorporating multiple mHealth functions [40,47]. In total, 3 studies used more than 1 mode of delivery, with ES varying across studies. A detailed description of the intervention’s effect on participants’ iron status is presented in Table 4.

### Secondary Outcomes

As presented in Table 5, 10 studies [15,40-48] reported at least 1 of the secondary outcomes relevant to this review. Among these 10 studies, 5 showed a statistically significant effect (*P*<.05) of mHealth intervention on adherence to iron and folic acid supplements in the intervention group, with adherence ranging from 63.8% [43] to 96% [48] in the intervention group.

**Table 5.** Summary of secondary outcomes.

Outcome categories and studies	Summary of findings
Adherence to IFAS^a^	
Elsharkawy et al [40]	Higher adherence to IFAS in the intervention group (90.8%) versus the control group (66.4%), *P*<.001
Sharma et al [43]	Higher adherence to IFAS in the intervention group (63.8%) versus the control group (39%), *P*=.001
Abujilban et al [45]	Higher mean IFAS adherence in the intervention group (mean 14.13, SD 2.68) versus the control group (mean 11.45, SD 3.02), *P*<.01
Washington et al [48]	Higher adherence to IFAS in the intervention group (96%) versus the control group (84%), *P*=.02
Gestational weight gain	
Singh et al [46]	Higher mean weight in the intervention group (mean 52.36, SD 6.28 kg) versus the control group (mean 50.94, SD 5.90 kg), *P*=.02
Wakwoya et al [15]	Slightly higher weight gain in the intervention group (9.69 kg) versus the control group (7.75 kg), but not statistically significant, *P*<.27
Xuto et al [42]	Higher mean weight gain in the intervention group (mean 12.77, SD 4.92 kg) versus the control group (mean 11.98, SD 6.42 kg), but not statistically significant, *P*=.60
Dietary intake	
Abd Rahman et al [47]	Higher mean dietary intake in the intervention group (mean 20.55, SD 5.12) versus the control group (mean 15.98, SD 3.68), *P*<.001
Wakwoya et al [15]	Intervention group consumed more: dark green vegetables (mean 17.96, SD 4.23 vs mean 10.37, SD 2.27; *P*<.001), vitamin C-rich foods (mean 5.42, SD 2.01 vs mean 4.20, SD 2.26; *P*=.005), eggs (mean 4.02, SD 1.04 vs mean 3.35, SD 1.10; *P*<.01), and meat, liver, and fish (mean 22.66, SD 4.77 vs mean 18.06, SD 3.36; *P*<.001) than the control group
Washington et al [48]	Intervention group consumed more fruits (91% vs 72%; aOR^b^ 3.77, 95% CI 1.49-9.54; *P*=.003), vegetables (96% vs 87%; aOR 3.69, 95% CI 0.97-14.00; *P*=.04), and fish, meat, or poultry (93% vs 83%; aOR 2.93, 95% CI 0.99-8.70; *P*=.04)
Nutritional knowledge	
Elsharkawy et al [40]	Higher mean knowledge score in intervention group (mean 54.33, SD 10.92) versus the control group (mean 24.79, SD 10.19), *P*<.001
Abd Rahman et al [47]	Higher mean knowledge score in intervention group (mean 26.41, SD 2.17) versus the control group (19.10, SD 3.44), *P*<.001
Abujilban et al [45]	Higher mean knowledge score in intervention group (mean 35.33, SD 9.55) versus the control group (mean 18.74, SD 9.55), *P*<.01
Attitude and practice	
—^c^	No included studies reported on this outcome
Implementation outcomes	
Sharma et al [44]	About 92.5% of pregnant women using the SwasthGarbh app expressed complete satisfaction

a IFAS: iron and folic acid supplement.

b aOR: adjusted odds ratio.

c Not available.

Among studies that evaluated the effect of mHealth intervention on gestational weight, only 1 study [46] reported a statistically significant association (*P*=.02). However, according to Wakwoya et al [15] and Xuto et al [42], although weight gain was slightly higher among pregnant women in the intervention group compared to the control group, the difference was not statistically significant (*P*=.27 and *P*=.60, respectively). Only 1 study [44] measured satisfaction levels among pregnant women adhering to the mHealth intervention. However, no studies provided data on maternal nutrition attitudes and practices or other patient-centered implementation outcomes, such as appropriateness, affordability, feasibility, acceptability, adoption, fidelity, sustainability, or the cost of mHealth intervention.

### Heterogeneity

Considerable heterogeneity was observed across studies, likely attributable to variance in participant characteristics (maternal age, gestational age, and Hb status), intervention features (mHealth delivery mode, mHealth interaction, and function), and socioeconomic and geographical contexts. Although a random-effects meta-analysis was conducted, heterogeneity was extreme *(I*^2^=95%; *χ*^2^_10_=196.7; *P*<.01), and the wide prediction interval (−0.92 to 2.82) restricted the interpretability of pooled effect estimates. Additionally, sensitivity analyses were performed to assess the influence of outlining studies, including those with large ESs and those reporting nonstatistically significant results. However, exclusion of these did not reduce heterogeneity, which remained high (*I*^2^>88%), nor did it substantially change the direction or statistical significance of the pooled effect. Therefore, we provided a narrative synthesis following guidance from the Cochrane Handbook for summarizing findings when meta-analysis was not feasible [23]

## Discussion

### Principal Findings

To the best of our knowledge, this is the first systematic review comprehensively assessing the effectiveness of mHealth-based nutritional interventions on maternal iron status. In this systematic review, 9 of 11 studies revealed a positive effect of mHealth-based nutritional intervention on Hb concentration during pregnancy. However, the ES varied from large ES (Cohen *d*>0.8) to small ES (Cohen *d*<0.5). Among the studies with a large ES, 3 used WhatsApp as an mHealth mode of delivery. These studies were conducted in high and upper-middle-income countries, where smartphone availability and access to data bundles are high, enabling mHealth interventions via WhatsApp platforms. In contrast, in LMICs, mHealth interventions mainly rely on SMS text messaging or telephone calls, as these delivery modes are more accessible through basic mobile devices [54,55]. The limited smartphone ownership, poor internet connectivity, and lower digital literacy in LMICs may contribute to reduced engagement with more interactive mHealth platforms.

According to a systematic review by Kante and Målqvist [56], the effectiveness of SMS-based interventions in LMICs varied across studies and settings, largely due to the differences in intervention type, content, frequency, and implementation approach. Some studies reported positive effects of SMS text messaging–based interventions on increasing the use of maternal and child health care services and improving adherence to iron supplementation among pregnant women [57,58]. These studies also identified SMS text messaging as a low-cost, easily personalized intervention that can be sent directly to target groups [57,58]. However, other studies found no significant effect of SMS text messaging on maternal health outcomes, including iron status during pregnancy [59].

Consistent with these findings, in our review, SMS text messaging was the most common mHealth delivery mode, used in 5 studies [15,42,43,46,48] conducted in LMICs. Despite its applicability, texting was associated with lower effectiveness than phone calls or WhatsApp-based interventions. This reduced effectiveness may be due to the limited educational content that can be conveyed via SMS text messaging and the lack of multimedia support, which could aid in information retention. Notably, the findings from this review suggest that combining SMS text messaging with other mHealth delivery modes, such as phone calls, significantly enhances intervention effectiveness. For instance, studies by Washington et al [48] and Sharma et al [43] reported ES of 1.61 (95% CI 1.25-1.99) and 0.79 (95% CI 0.53-1.04), respectively, when SMS text messaging was combined with phone calls.

Moreover, a study suggested that combining SMS text messaging with an interactive feature could improve the effectiveness of mHealth interventions [59]. Similarly, the highest ES (2.61, 95% CI 2.23-3.00) in this review was observed in a study that used text-based education via WhatsApp, supplemented by interactive features or 2-way communication [40].

In total, 3 studies [40,47,48] demonstrating the highest ES also showed clinically significant improvements in mean Hb concentration, with differences greater than 1 g/dL within- and between-group comparisons. These studies conveyed nutritional advice through different delivery modes, in which 2 studies [40,48] reported higher adherence to iron and folic acid supplements, and the third study [47] found higher dietary iron intake among intervention groups adhering to mHealth-supported nutrition interventions. Furthermore, the mean Hb concentrations observed in these 3 studies were notably higher than the pooled results from 2 meta-analyses, which found mean Hb differences of 0.46 and 0.89 g/dL in pregnant women taking iron with or without folic acid, respectively [60,61]. Similarly, a review by Engidaw et al [16] reported a mean Hb difference of 0.88 g/dL based on pooled results from 39 studies, of which only 8 used an mHealth-based approach. While it is acknowledged that comparing individual study results with pooled meta-analysis data has limitations, the higher mean Hb concentration difference observed in this review may be attributed to the nutritional interventions supported by mHealth technologies. This technological support may have improved participants’ adherence to the interventions, thereby enhancing the intervention outcomes.

All 11 studies included in this review focused on nutrition-specific interventions, which address the most proximal causes of anemia, such as dietary intake and iron and folic acid supplementation. Nonetheless, 5 studies [15,44,46,48,49] incorporated nutrition-sensitive interventions, which address underlying determinants of anemia, such as maternal health care access and hygiene practices. Notably, this review found that studies incorporating nutrition-sensitive components did not demonstrate higher ES compared to those focusing solely on nutrition-specific interventions. This may be due to 2 key factors. First, the complex and multifactorial etiology of anemia may hinder the direct effects of nutrition-sensitive interventions, especially within short study time frames [6]. Second, mHealth platforms may not be optimally suited for delivering certain nutrition-sensitive interventions, such as maternal reproductive health services that require in-depth counseling, physical demonstrations by skilled health care providers, or access to physical resources [62].

Additionally, the meta-review by Moorthy et al [13] synthesized data from 118 systematic reviews to assess the impact of nutritional interventions on Hb concentration and anemia outcomes in specific populations, including pregnant women. This comprehensive analysis highlighted the effectiveness of key nutrition-specific interventions, such as iron and folic acid supplementation and intermittent preventive treatment in pregnancy for malaria, in optimizing Hb concentration among pregnant women. However, only 5 of the 118 systematic reviews focused on the impact of nutrition-sensitive interventions on Hb concentration and anemia prevalence, indicating a knowledge gap in the evidence [13]. This paucity of data constrains our ability to draw robust conclusions about the effectiveness of nutrition-sensitive interventions in enhancing maternal iron status.

The effectiveness of mHealth-based nutritional interventions may also rely on the integration of BCTs, which refer to the specific techniques used in the intervention to promote behavior change [63,64]. However, despite their broad use in health promotion, only 3 (27.3%) studies in this review explicitly used existing behavior change theories such as the health belief model or the theory of planned behavior. Notably, one study incorporating the health belief model reported a small ES (ES=0.29, 95% CI 0.07-0.51), while another study with the largest ES (ES=2.61, 95% CI 2.23-3.00) did not apply any behavior change theories [15,40]. This discrepancy suggests that the effectiveness of mHealth interventions may not rely solely on the inclusion of a single behavior change theory or BCTs but rather on the integration of diverse theories or techniques alongside optimal mHealth delivery modes [64,65]. These findings align with systematic reviews, emphasizing that combining multiple BCTs, such as feedback and monitoring, prompts and cues, personalization, and goals and planning, enhances intervention outcomes [66,67].

### Limitations of the Review

A key limitation of this review is the inability to include a meta-analysis due to substantial heterogeneity across studies, including variations in mHealth delivery modes, intervention content, and types of nutritional interventions. As a result, a formal statistical assessment of publication bias (eg, funnel plot analysis) could not be conducted. Nevertheless, studies with null or nonsignificant findings may be underrepresented due to publication bias.

This review was restricted to peer-reviewed studies published in English, potentially leading to language and publication bias by excluding relevant evidence from the gray literature. Additionally, interrater reliability statistics (eg, Cohen κ) were not calculated, as data extraction was conducted by only 1 reviewer (SAB).

Furthermore, many of the included studies (90.9%) were judged to have some concerns regarding the risk of bias, potentially limiting the quality of evidence. Finally, this review did not focus on mHealth interventions designed for health care providers, which may have influenced maternal health outcomes indirectly by enhancing maternal nutrition care and adherence to antenatal guidelines.

### Conclusions

This systematic review demonstrates that mHealth-supported nutritional interventions can effectively optimize Hb concentrations in pregnant women. Interventions using WhatsApp showed the most significant impact, potentially due to their capacity to deliver multimedia-rich content, thereby facilitating better information retention. Conversely, interventions relying solely on SMS text messaging were less effective; however, combining SMS text messaging with other delivery modes, such as phone calls, improved overall effectiveness.

The integration of mHealth interventions into maternal health care for anemia prevention and management is both feasible and supported by evidence. Nevertheless, the variability in mHealth delivery modes, functions, and interactive features underscores the need for tailored strategies that consider context-specific challenges, digital literacy levels, and access to technology to increase effectiveness.

### Implications for Practice and Future Research

The current evidence is predominantly generated from studies conducted in a limited number of countries, which constrains the generalizability of findings. Future research should focus on evaluating the effectiveness of mHealth-based nutritional interventions on maternal iron status across diverse geographical and socioeconomic contexts to strengthen the applicability of the conclusions. Additionally, the insights from this review can guide future researchers in understanding the impact of various mHealth delivery modes, functions, and interactive features on the iron status of pregnant women. Such understanding will assist mHealth intervention designers and implementation researchers in adopting and developing context-specific approaches and identifying implementation strategies to improve effectiveness.

Given the importance of patient-centered care, mHealth interventions must be designed with a personalized approach. Thus, future researchers should also emphasize evaluating implementation outcomes to ensure the effectiveness and sustainability of mHealth interventions in real-world settings.

## Supplementary material

10.2196/81001 Multimedia Appendix 1 Search strategy used for PubMed (including MEDLINE via NCBI), Embase, Web of Science Core Collection, Scopus, CENTRAL (via Cochrane Library), and CINAHL (via EBSCOhost), combining terms related to mobile health, nutritional interventions, iron status, and pregnant women.

10.2196/81001 Multimedia Appendix 2 Eligibility criteria used to define the research question.

10.2196/81001 Multimedia Appendix 3 Risk of bias assessment for randomized controlled trials.

10.2196/81001 Checklist 1 PRISMA checklist.

## Abbreviations

BCT: behavior change technique
cRCT: cluster randomized controlled trial
ES: effect size
Hb: hemoglobin
LMIC: low- and middle-income countries
mHealth: mobile health
PRISMA: Preferred Reporting Items for Systematic Reviews and Meta-Analyses
PROSPERO: International Prospective Register of Systematic Reviews
RCT: randomized controlled trial
