# Supplementary material for: Effectiveness of mHealth-Based Nutritional Interventions on Iron Status of Pregnant Women: Systematic Review of Randomized Controlled Trials
Source: JMIR Mhealth Uhealth. 2026 Apr 9;14:e81001. doi: 10.2196/81001 (PMC13065237; doi:10.2196/81001)
Supplement: Multimedia Appendix 1 [file mhealth-v14-e81001-s001.docx]

**PubMed (including Medline, via NCBI)**

"Telemedicine"[Mesh] OR “telemedicine”[tiab] OR “Virtual*”[tiab] OR “Mobile Health*”[tiab] OR “mHealth*”[tiab] OR “m-Health*”[tiab] OR “Telehealth*”[tiab] OR “Tele-health*”[tiab] OR “eHealth*”[tiab] OR “e-Health*”[tiab] OR “Telecare”[tiab] OR “Tele-Care”[tiab] OR “e-care”[tiab] OR “distance counseling”[tiab] OR “e-counseling”[tiab] OR “online counseling”[tiab] OR “remote counseling”[tiab] OR “tele counseling”[tiab] OR “e-therap*”[tiab] OR "Cell Phone"[Mesh] OR “cell phone*”[tiab] OR “cellular phone*”[tiab] OR “cellular telephone*”[tiab] OR “mobile phone*”[tiab] OR “mobile telephone*”[tiab] OR “portable phone*”[tiab] OR “portable telephone*”[tiab] OR “transportable phone”[tiab:~0] OR “transportable phones”[tiab:~0] OR “transportable telephone”[tiab:~0] OR “transportable telephones”[tiab:~0] OR “smartphone*”[tiab] OR “smart phone*”[tiab] OR “text messag*”[tiab] OR “texting”[tiab] OR “short message service”[tiab] OR “SMS”[tiab] OR “'short messaging service” OR “phone call*”[tiab] OR “calling”[tiab] OR "Social Media"[Mesh] OR “social media”[tiab] OR "Wireless Technology"[Mesh] OR “Wireless Technolog*”[tiab] OR "Internet-Based Intervention"[Mesh] OR “Internet-Based Intervention*"[tiab] AND "Prenatal Care"[Mesh] OR “Prenatal Care” [tiab] OR “Pre-natal Care” [tiab] OR “prenatal screening” [tiab] OR “antenatal care”[tiab] OR “ante natal care”[tiab] OR "Diet, Food, and Nutrition"[Mesh] OR “Prenatal Nutritional Physiological Phenomena"[Mesh] OR “nutrition*”[tiab] OR "Nutrients"[Mesh] OR “nutrient*” OR “diet*”[tiab] OR “food*”[tiab] OR “iron”[tiab] OR “gestational weight gain*”[tiab] OR “pregnancy weight gain”[tiab] OR “supplement*”[tiab] OR “IFA”[tiab] OR “vitamin*”[tiab] OR “folate”[tiab] OR “Folic acid”[tiab] OR “meal*”[tiab] OR “eat”[tiab] OR “eating”[tiab] OR “bio-fortificat*”[tiab] AND "Hemoglobins"[Mesh:NoExp] OR Hemoglobin*[tiab] OR Haemoglobin*[tiab] OR “Hb”[tiab] OR "Anemia"[Mesh] OR Anemia*[tiab] OR “anaemia*”[tiab] OR "Iron Deficiencies"[Mesh] OR "Anemia, Iron-Deficiency"[Mesh] OR "Iron Metabolism Disorders"[Mesh:NoExp] OR "Ferritins"[Mesh:NoExp] OR "Ferritin*"[tiab] AND "Pregnant Women"[Mesh] OR "Pregnancy"[Mesh] OR “pregnan*” [tiab] OR “gestation”[tiab] OR “expectant mother*”[tiab] OR “childbearing”[tiab] OR “gravid*”[tiab] OR “prenatal*”[tiab] OR “pre-natal*”[tiab] OR “antenatal*”[tiab] OR “ante-natal*”[tiab]

**Embase (via Embase.com)**

'telemedicine':ti,ab,kw OR 'virtual*':ti,ab,kw OR 'mobile health application'/exp OR 'mobile health*':ti,ab,kw OR 'mhealth'/exp OR 'mhealth*':ti,ab,kw OR 'm-health*':ti,ab,kw OR 'telehealth'/exp OR 'telehealth*':ti,ab,kw OR 'tele-health*':ti,ab,kw OR 'ehealth*':ti,ab,kw OR 'e-health*':ti,ab,kw OR 'telecare':ti,ab,kw OR 'tele-care':ti,ab,kw OR 'e-care':ti,ab,kw OR 'e-counseling'/exp OR 'e-counseling':ti,ab,kw OR 'distance counseling':ti,ab,kw OR 'online counseling':ti,ab,kw OR 'remote counseling':ti,ab,kw OR 'tele counseling':ti,ab,kw OR 'mobile phone'/exp OR 'mobile phone*':ti,ab,kw OR 'cell phone*':ti,ab,kw OR 'cellular phone*':ti,ab,kw OR 'cellular telephone*':ti,ab,kw OR 'mobile telephone*':ti,ab,kw OR 'smartphone'/exp OR 'smartphone*':ti,ab,kw OR 'smart phone*':ti,ab,kw OR 'phone call'/exp OR 'phone call*':ti,ab,kw OR 'calling':ti,ab,kw OR 'text messaging'/exp OR 'text messag*':ti,ab,kw OR 'texting':ti,ab,kw OR 'short message service'/exp OR 'short message service':ti,ab,kw OR 'short messaging service'/exp OR 'short messaging service*':ti,ab,kw OR 'SMS':ti,ab,kw OR 'portable phone*':ti,ab,kw OR 'portable telephone*':ti,ab,kw OR 'transportable phone*':ti,ab,kw OR 'transportable telephone*':ti,ab,kw OR 'social media'/exp OR 'social media':ti,ab,kw OR 'wireless technolog*':ti,ab,kw OR 'Internet-Based Intervention*':ti,ab,kw AND 'nutritional intervention'/exp OR 'prenatal care'/de OR ‘prenatal screening’/exp OR ‘prenatal screening’:ti,ab,kw OR 'prenatal care':ti,ab,kw OR ‘pre-natal care’:ti,ab,kw OR 'antenatal care':ti,ab,kw OR 'ante natal care':ti,ab,kw OR 'nutrition'/exp OR 'nutrition*':ti,ab,kw OR 'nutrient'/exp OR ‘nutrient*’:ti,ab,kw OR 'diet*':ti,ab,kw OR 'food*':ti,ab,kw OR 'iron':ti,ab,kw OR 'gestational weight gain'/exp OR 'gestational weight gain':ti,ab,kw OR 'pregnancy weight gain':ti,ab,kw OR 'supplementation'/exp OR 'supplement*':ti,ab,kw OR 'IFA':ti,ab,kw OR 'vitamin'/exp OR 'vitamin*':ti,ab,kw OR 'biofortification'/exp OR 'bio-fortificat*':ti,ab,kw OR 'folic acid'/exp OR 'folic acid':ti,ab,kw OR 'meal'/exp OR 'meal*':ti,ab,kw OR 'eating'/exp OR 'eat*':ti,ab,kw AND 'hemoglobin'/de OR 'hemoglobin*':ti,ab,kw OR 'haemoglobin*':ti,ab,kw OR ‘Hb’:ti,ab,kw OR 'anemia'/exp OR 'anemia*':ti,ab,kw OR 'anaemia*':ti,ab,kw OR 'iron deficiency anemia'/exp OR 'iron metabolism disorder*':ti,ab,kw OR 'iron metabolism defect*':ti,ab,kw OR 'ferritin'/exp OR 'ferritin*':ti,ab,kw AND 'pregnant woman'/exp OR 'pregnancy'/exp OR 'pregnan*':ti,ab,kw OR 'gestation':ti,ab,kw OR 'childbearing':ti,ab,kw OR 'gravid*':ti,ab,kw OR 'expectant mother'/exp OR 'expectant mother*':ti,ab,kw OR ‘prenatal*’:ti,ab,kw OR ‘pre-natal*’:ti,ab,kw OR ‘antenatal*’:ti,ab,kw OR ‘ante-natal*’:ti,ab,kw

**Web of Science core collection (Via web of science.com)**

TS=(“telemedicine” OR “virtual*” OR “mobile health*” OR “mhealth*” OR “m-health*” OR “telehealth*” OR “tele-health*” OR “ehealth*” OR “e-therap*” OR “e-health*” OR “telecare” OR “tele-care” OR “e-care” OR “e-counseling” OR “distance counseling” OR “online counseling” OR “remote counseling” OR “tele counseling” OR “mobile phone*” OR “cell phone*” OR “cellular phone*” OR “cellular telephone*” OR “mobile telephone*” OR “smartphone*” OR “smart phone*” OR “phone call*” OR “calling” OR “text messag*” OR “texting” OR “short message service” OR “sms” OR “short messaging service” OR “portable phone*” OR “portable telephone*” OR “transportable phone*” OR “transportable telephone*” OR “social media” OR "Wireless Technolog*" OR “Internet-Based Intervention*”) AND TS=(“prenatal care” OR “pre-natal care” OR “prenatal screening” OR “antenatal care” OR “ante natal care” OR “prenatal nutritional physiological phenomena” OR “nutrition*” OR “nutrient*” OR “diet*” OR “food*” OR “iron” OR “gestational weight gain*” OR “pregnancy weight gain” OR “supplement*” OR “IFA” OR “vitamin*” OR “folate” OR “folic acid” OR “meal*” OR “eat*” OR “eating” OR “bio-fortificat*”) AND TS=(“hemoglobin*” OR “haemoglobin*” OR “Hb” OR “anemia” OR “anaemia” OR “iron deficiency anemia” OR “anemia, iron-deficiency” OR “iron-deficiency anemia” OR “iron metabolism disorder*” OR “iron metabolism defect*” OR “ferritin*”) AND TS=(“pregnant wom*” OR “pregnan*” OR “gestation” OR “childbearing” OR “gravid*” OR “expectant mother*” OR “prenatal*” OR “pre-natal*” OR “antenatal*” OR “ante-natal*”)

**Scopus (via scopus.com)**

TITLE-ABS ("telemedicine" OR "virtual*" OR "mobile health*" OR "mhealth*" OR "m-health*" OR "telehealth*" OR "tele-health*" OR "ehealth*" OR "e-therap*" OR "e-health*" OR "telecare" OR "tele-care" OR "e-care" OR "e-counseling" OR "distance counseling" OR "online counseling" OR "remote counseling" OR "tele counseling" OR "mobile phone*" OR "cell phone*" OR "cellular phone*" OR "cellular telephone*" OR "mobile telephone*" OR "smartphone*" OR "smart phone*" OR "phone call*" OR "calling" OR "text messag*" OR "texting" OR "short message service" OR "sms" OR “short messaging service” OR "portable phone*" OR "portable telephone*" OR "transportable phone*" OR "transportable telephone*" OR "social media" OR "Wireless Technolog*" OR “Internet-Based Intervention*”) OR AUTHKEY("telemedicine" OR "virtual*" OR "mobile health*" OR "mhealth*" OR "m-health*" OR "telehealth*" OR "tele-health*" OR "ehealth*" OR "e-therap*" OR "e-health*" OR "telecare" OR "tele-care" OR "e-care" OR "e-counseling" OR "distance counseling" OR "online counseling" OR "remote counseling" OR "tele counseling" OR "mobile phone*" OR "cell phone*" OR "cellular phone*" OR "cellular telephone*" OR "mobile telephone*" OR "smartphone*" OR "smart phone*" OR "phone call*" OR "calling" OR "text messag*" OR "texting" OR "short message service" OR "sms" OR “short messaging service” OR "portable phone*" OR "portable telephone*" OR "transportable phone*" OR "transportable telephone*" OR "social media" OR "Wireless Technolog*" OR “Internet-Based Intervention*”) AND TITLE-ABS (“prenatal care” OR “pre-natal care” OR “prenatal screening” OR “antenatal care” OR “ante natal care” OR “prenatal nutritional physiological phenomena” OR “nutrition*” OR “nutrient*” OR “diet*” OR “food*” OR “iron” OR “gestational weight gain*” OR “pregnancy weight gain” OR “supplement*” OR “IFA” OR “vitamin*” OR “folate” OR “folic acid” OR “meal*” OR “eat*” OR “eating” OR “bio-fortificat*”) OR AUTHKEY(“prenatal care” OR “pre-natal care” OR “prenatal screening” OR “antenatal care” OR “ante natal care” OR “diet, food, and nutrition” OR “prenatal nutritional physiological phenomena” OR “nutrition*” OR “diet*” OR “food*” OR “iron” OR “gestational weight gain*” OR “pregnancy weight gain” OR “supplement*” OR “IFA” OR “vitamin*” OR “folate” OR “folic acid” OR “meal*” OR “eat*” OR “eating” OR “bio-fortificat*”) AND TITLE-ABS(“hemoglobin*” OR “haemoglobin*” OR “Hb” OR “anemia” OR “anaemia” OR “iron deficiency anemia” OR “anemia, iron-deficiency” OR “iron-deficiency anemia” OR “iron metabolism disorder*” OR “iron metabolism defect*” OR “ferritin*”) OR AUTHKEY(“hemoglobin*” OR “haemoglobin*” OR “Hb” OR “anemia” OR “anaemia” OR “iron deficiency anemia” OR “anemia, iron-deficiency” OR “iron-deficiency anemia” OR “iron metabolism disorder*” OR “iron metabolism defect*” OR “ferritin*”) AND TITLE-ABS (“pregnant wom*” OR “pregnan*” OR “gestation” OR “childbearing” OR “gravid*” OR “expectant mother*” OR “prenatal*” OR “pre-natal*” OR “antenatal*” OR “ante-natal*”) OR AUTHKEY(“pregnant wom*” OR “pregnan*” OR “gestation” OR “childbearing” OR “gravid*” OR “expectant mother*” OR “prenatal*” OR “pre-natal*” OR “antenatal*” OR “ante-natal*”)

**CENTRAL (via Cochrane Library)**

**#1** [mh "Telemedicine"]

# 2: [mh "Cell Phone"]

#3: [mh "wireless technology"]

# 4: [mh "Internet-Based Intervention"]

# 5: [mh "social media"]

# 6: ("telemedicine" OR Virtual* OR (Mobile NEXT Health*) OR mHealth* OR (m NEXT Health*) OR Telehealth* OR (Tele NEXT health*) OR eHealth* OR (e NEXT Health*) OR "Telecare" OR "Tele-Care" OR "e-care" OR "distance counseling" OR "e-counseling" OR "online counseling" OR "remote counseling" OR "tele counseling" OR (e NEXT therap*) OR (cell NEXT phone*) OR (cellular NEXT phone*) OR (cellular NEXT telephone*) OR (mobile NEXT phone*) OR (mobile NEXT telephone*) OR (portable NEXT phone*) OR (portable NEXT telephone*) OR (transportable NEXT phone*) OR (transportable NEXT telephone*) OR (smart NEXT phone*) OR (text NEXT messag*) OR "texting" OR "short message service" OR "SMS" OR "short messaging service" OR (phone NEXT call*) OR "calling" OR "social media" OR (Wireless NEXT Technolog*) OR ("Internet-Based" NEXT Intervention*)):ti,ab,kw AND

#7: **#1** AND # 2 AND #3 AND #4 AND #5 AND #5 AND #6

**#8: [**mh "Prenatal Care"]

#9: [mh “Prenatal Nutritional Physiological Phenomena"]

#10: [ mh “nutrients”]

**#11:** ("Prenatal Care" OR "Pre-natal Care" OR "prenatal screening" OR "antenatal care" OR "ante natal care" OR nutrition* OR nutrient* OR diet* OR food* OR "iron" OR ("gestational weight" NEXT gain*) OR "pregnancy weight gain" OR supplement* OR "IFA" OR vitamin* OR "folate" OR "Folic acid" OR meal* OR "eat" OR "eating" OR ("bio NEXT fortificat*")):ti,ab,kw AND

**#13: [**mh ^"Hemoglobins"]

#14: **[**mh "Anemia"]

#15: **[**mh "Iron Deficiencies"]

#16:**[**mh "Anemia, Iron-Deficiency"]

#17: **[**mh ^"Iron Metabolism Disorders"]

#18: **[**mh ^"Ferritins"]

#19: (Hemoglobin* OR Haemoglobin* OR “Hb” OR Anemia* OR anaemia* OR Ferritin*):ti,ab,kw AND

#20: #7 AND #8 AND #9 AND #10 AND #11 AND #12 AND #13 AND #14 AND #15 AND #16 AND #17 AND #18 AND #19

**#21: [**mh "Pregnant Women"]

#22: **[**mh "Pregnancy"]

#23: (pregnan* OR "gestation" OR (expectant NEXT mother*) OR "childbearing" OR gravid* OR prenatal* OR (pre NEXT natal*) OR antenatal* OR (ante NEXT natal*)):ti,ab,kw

#24: #20 AND #21 AND #22 AND #23

**CINAHL (via EBSCOhost)**

(MH "Telemedicine+") OR (MH "Telehealth") OR (MH "Cellular Phone+") OR (MH "Mobile Applications") OR (MH "Text Messaging") OR (MH "Social Media+") OR (MH "Internet-Based Intervention") OR TI (“telemedicine” OR “virtual*” OR “mobile health*” OR “mhealth*” OR “m-health*” OR “telehealth*” OR “tele-health*” OR “ehealth*” OR “e-health*” OR “telecare” OR “tele-care” OR “e-care” OR “e-counseling” OR “distance counseling” OR “online counseling” OR “remote counseling” OR “tele counseling” OR “mobile phone*” OR “cell phone*” OR “cellular phone*” OR “cellular telephone*” OR “mobile telephone*” OR “smartphone*” OR “smart phone*” OR “phone call*” OR “calling” OR “text messag*” OR “texting” OR “short message service” OR “short messaging service*” OR “sms” OR “portable phone*” OR “portable telephone*” OR “transportable phone*” OR “transportable telephone*” OR “Social media” OR "Internet-Based Intervention*" OR "Wireless Technolog*") OR AB (“telemedicine” OR “virtual*” OR “mobile health*” OR “mhealth*” OR “m-health*” OR “telehealth*” OR “tele-health*” OR “ehealth*” OR “e-health*” OR “telecare” OR “tele-care” OR “e-care” OR “e-counseling” OR “distance counseling” OR “online counseling” OR “remote counseling” OR “tele counseling” OR “mobile phone*” OR “cell phone*” OR “cellular phone*” OR “cellular telephone*” OR “mobile telephone*” OR “smartphone*” OR “smart phone*” OR “phone call*” OR “calling” OR “text messag*” OR “texting” OR “short message service” OR “short messaging service*” OR “sms” OR “portable phone*” OR “portable telephone*” OR “transportable phone*” OR “transportable telephone*” OR “Social media” OR "Internet-Based Intervention*" OR "Wireless Technolog*") AND

(MH "Prenatal Care") OR (MH "Prenatal Nutritional Physiology") OR (MH "Nutrition+") OR (MH "Diet+") OR (MH "Food+") OR (MH "Iron") OR (MH "Gestational Weight Gain") OR (MH "Dietary Supplementation") OR (MH "Dietary Supplements") OR (MH "Vitamins+") OR (MH "Folic Acid") OR (MH "Meals") OR (MH "Eating") OR TI (“Prenatal Care” OR “Pre-natal Care” OR “prenatal screening” OR “antenatal care” OR “ante natal care” OR “Prenatal Nutritional Physiological Phenomena" OR “nutrition*” OR “nutrient*” OR “diet*” OR “food*” OR “iron” OR “gestational weight gain*” OR “pregnancy weight gain” OR “supplement*” OR “IFA” OR “vitamin*” OR “folate” OR “Folic acid” OR “meal*” OR “eat” OR “eating” OR “bio-fortificat*”) OR AB (“Prenatal Care” OR “Pre-natal Care” OR “prenatal screening” OR “antenatal care” OR “ante natal care” OR “Prenatal Nutritional Physiological Phenomena" OR “nutrition*” OR “diet*” OR “food*” OR “iron” OR “gestational weight gain*” OR “pregnancy weight gain” OR “supplement*” OR “IFA” OR “vitamin*” OR “folate” OR “Folic acid” OR “meal*” OR “eat” OR “eating” OR “bio-fortificat*”) AND (MH "Hemoglobins") OR (MH "Anemia") OR (MH "Iron Deficiencies") OR (MH "Anemia, Iron Deficiency") OR (MH "Iron Metabolism Disorders") OR (MH "Ferritin") OR TI (“Hemoglobin*” OR “Haemoglobin*” OR “Hb” OR "Anemia" OR “anaemia*” OR "Iron Deficiencies" OR "Anemia, Iron-Deficiency" OR "Iron Metabolism Disorders" OR "Ferritin*") OR AB (“Hemoglobin*” OR “Haemoglobin*” OR “Hb” OR "Anemia" OR “anaemia*” OR "Iron Deficiencies" OR "Anemia, Iron-Deficiency" OR "Iron Metabolism Disorders" OR "Ferritin*") AND (MH "Expectant Mothers") OR (MH "Pregnancy") OR TI ("Pregnant Women" OR "Pregnancy” OR “pregnan*” OR “gestation” OR “expectant mother*” OR “childbearing” OR “gravid*” OR “prenatal*” OR “pre-natal*” OR “antenatal*” OR “ante-natal*”) OR AB ("Pregnant Women" OR "Pregnancy” OR “pregnan*” OR “gestation” OR “expectant mother*” OR “childbearing” OR “gravid*” OR “prenatal*” OR “pre-natal*” OR “antenatal*” OR “ante-natal*”)
