# Supplementary material for: Effectiveness of mHealth-Based Nutritional Interventions on Iron Status of Pregnant Women: Systematic Review of Randomized Controlled Trials
Source: JMIR Mhealth Uhealth. 2026 Apr 9;14:e81001. doi: 10.2196/81001 (PMC13065237; doi:10.2196/81001)
Supplement: Multimedia Appendix 2 [file mhealth-v14-e81001-s002.docx]

**Table S1.** Eligibility criteria used to define the research question.

| Attribute | Inclusion criteria | Exclusion criteria |
| --- | --- | --- |
| Population | Pregnant women | Mixed populations (both pregnant and non-pregnant women) |
| Intervention | mHealth interventions focusing on maternal nutrition as an intervention or part of an intervention package. | Routine ANC without mHealth and mHealth interventions targeting HCPs |
| Outcome | Iron status described by Hb levels (g/dL) | Studies did not assess iron status |
| Comparison | Standard maternal nutritional interventions (without mHealth) delivered in ANC | NA |
| Types of studies | Peer-reviewed Randomized Controlled Trials | Observational studies (cohort, case-control, cross-sectional, and qualitative studies), case reports, reviews, project/program reports, conference proceedings |
| Language | Studies available in the English Language | Studies which were not available in English translation |
| Period | Studies published between January 1^st^, 2003 and December 30^th^, 2024 (the date that the last searches were conducted) | Studies published before 1 January 2003 |
| Study setting | Studies conducted or implemented in any country. | NA |

NA: Not applicable.
