# Supplementary material for: Effectiveness of mHealth-Based Nutritional Interventions on Iron Status of Pregnant Women: Systematic Review of Randomized Controlled Trials
Source: JMIR Mhealth Uhealth. 2026 Apr 9;14:e81001. doi: 10.2196/81001 (PMC13065237; doi:10.2196/81001)

Risk of bias assessment for Randomized Control Trials

| Study | Domain 1 | | Domain 2 | Domain 3 | Domain 4 | Domain 5 |  |
| --- | --- | --- | --- | --- | --- | --- | --- |
|  | Allocation sequence generation. | Allocation sequence concealment. | Bia due to deviations from intended interventions | Bis due to missing outcome data. | Bias in measurement of the outcome. | Bias in the selection of the reported result. | Overall Judgment |
| Elsharkawy et al [40] | Judgment: | Judgment: | Judgment: | Judgment: | Judgment: | Judgment: |  |
|  | Support:  A random sequence allocation was performed using a permutation block randomization. | Support:  Allocation was concealed in sealed, opaque envelopes. | Support:  Only the participants were blinded to the intervention. However, no deviations from the intended intervention were observed. | Support:  No loss to follow-up was observed, and the analysis was performed based on the intention-to-treat principle. | Support:  The outcome measurement was appropriate. It is unclear whether outcome assessors were blinded, but Hb measurement is unlikely to be influenced by a lack of blinding. | Support:  The authors reported the expected outcomes  per pre-specified plan. | **Low risk of bias** |
| Sontakke et al [41] | Judgment: | Judgment: | Judgment: | Judgment: | Judgment: | Judgment: |  |
|  | Support:  A random sequence allocation was performed using a computer-generated simple randomization. | Support:  The method used for allocation sequence concealment was not reported. | Support:  There was no report on blinding or how deviations from the intended intervention were minimized. | Support:  Approximately 13 participants (5.1%) were lost to follow-up in the intervention group, and the reasons for dropout were described. | Support:  The outcome measurement was appropriate. It is unclear whether outcome assessors were blinded, but Hb measurement is unlikely to be influenced by a lack of blinding. | Support:  The authors reported the study's outcomes, but it is unclear whether this was per the pre-specified plan. Neither study protocol nor proof of Clinical Trail Registry available.  Additionally, more frequent Hb estimations were performed, which may introduce reporting bias. | **Some**  **Concerns** |
| Singh et al [46] | Judgment: | Judgment: | Judgment: | Judgment: | Judgment: | Judgment: |  |
|  | Support:  A random sequence allocation was performed using a simple random number sequence. | Support:  A method used for allocation sequence concealment was not reported. | Support:  Neither trial personnel nor participants were blinded due to the nature of the study; however, no deviations from the intended intervention were observed. | Support:  A low attrition rate (1.9%) was reported in both the intervention group (n=5) and the control group (n=8), with reasons for loss to follow-up clearly described. | Support:  The outcome measurement was appropriate. It is unclear whether outcome assessors were blinded, but Hb measurement is unlikely to be influenced by a lack of blinding. | Support:  The authors reported the study's outcomes, but it is unclear whether this was per the pre-specified study protocol. | **Some**  **Concerns** |
| Abd Rahman et al [47] | Judgment: | Judgment: | Judgment: | Judgment: | Judgment: | Judgment: | : |
|  | Support:  A random sequence allocation was performed using a random number generator. | Support:  A method used for allocation sequence concealment was not reported. | Support:  Only the participants were blinded to the intervention; however, no deviations from the intended intervention were observed. | Support:  Approximately 16 participants (13.3%) were lost to follow-up across the intervention group (n=6) and control group (n=10), with reasons for loss to follow-up clearly described. | Support:  The outcome measurement was appropriate. It is unclear whether outcome assessors were blinded, but Hb measurement is unlikely to be influenced by a lack of blinding. | Support:  The authors reported the study's outcomes, but it is unclear whether this was per the pre-specified study protocol. | **Some**  **Concerns** |
| Xuto et al [42] | Judgment: | Judgment: | Judgment: | Judgment: | Judgment: | Judgment: |  |
|  | Support:  A random sequence allocation was performed using a random number table | Support:  The method used for allocation sequence concealment was not reported. | Support:  Only the participants were blinded to the intervention. However, no deviations from the intended intervention were observed. | Support:  Approximately 7 participants (10.6%) were lost to follow-up, with 3 from the intervention group and 4 from the control group. The reasons for dropout were described. | Support:  There was no report on how Hb measurements were taken, and it is unclear whether outcome assessors were blinded. | Support:  The authors reported the expected outcomes  per the pre-specified plan. Clinical Trial Registry Number available. | **Some**  **Concerns** |
| Sharma et al [44] | Judgment: | Judgment: | Judgment: | Judgment: | Judgment: | Judgment: |  |
|  | Support:  A random sequence allocation was performed using a computer-generated random allocation | Support:  The method used for allocation sequence concealment was not reported. | Support:  Neither trial personnel nor participants were blinded due to the nature of the study; however, no deviations from the intended intervention were observed. | Support:  Approximately 7 participants (4.7%) were lost to follow-up, with 4 from the intervention group and 3 from the control group. The reasons for dropout were described. | Support:  There was no report on how Hb measurements were taken, and it is unclear whether outcome assessors were blinded. | Support:  The authors reported the expected outcomes  per the pre-specified plan. Clinical Trial Registry Number is available. | **Some**  **Concerns** |
| Washington et al [48] | Judgment: | Judgment: | Judgment: | Judgment: | Judgment: | Judgment: |  |
|  | Support:  The method used to generate the random sequence allocation was not reported. | Support:  The method used for allocation sequence concealment was not reported. | Support:  Participants, investigators, and data collectors were unaware of the intervention. | Support:  Only three participants (2%) were lost to follow-up due to withdrawal in the control group, but they were included in the analysis using the intent-to-treat approach. | Support:  The outcome measurement was appropriate. Outcome assessors were aware of the trial, but Hb measurement is unlikely to be influenced by a lack of blinding. | Support:  The authors reported the study outcomes. No study protocol available but outcomes outlined in Clinical Trail Registry were reported. | **Some**  **Concerns** |
| Wakwoya et al [15] | Judgment: | Judgment: | Judgment: | Judgment: | Judgment: | Judgment: |  |
|  | Support:  A random sequence allocation was performed using a random number table generator. | Support:  A data manager who was not a member of the research team randomly allocated clusters to the intervention and control arms. | Support:  Participants, counselors, and data collectors were blinded to the intervention. | Support:  Approximately 40 participants (10.9%) were lost to follow-up, 18 from the intervention group and 20 from the control group. The reasons for loss to follow-up were described, and a proper follow-up protocol was implemented. | Support:  The outcome measurement was appropriate. The outcome assessors were aware of the trial, but Hb measurement is unlikely to be influenced by a lack of blinding. | Support:  The authors reported the study's outcomes, but it is unclear whether this was per the pre-specified plan.  Neither the study protocol nor proof of the Clinical Trail Registry is available. | **Some**  **Concerns** |
| Ahmad et al [49] | Judgment: | Judgment: | Judgment: | Judgment: | Judgment: | Judgment: |  |
|  | Support:  The method used to generate a random sequence allocation was not reported. | Support:  The method used for allocation sequence concealment was not reported. | Support:  There was no report on blinding or how deviations from the intended intervention were minimized. | Support:  There was no report on loss to follow-up or how missing data was handled. | Support:  The outcome measurement was appropriate. It is unclear whether outcome assessors were blinded, but Hb measurement is unlikely to be influenced by a lack of blinding | Support:  The authors reported the expected outcomes, but it is unclear whether they align with the pre-specified plan. Neither study protocol nor proof of Clinical Trail Registry available. | **Some**  **Concerns** |
| S. Sharma et al [43] | Judgment: | Judgment: | Judgment: | Judgment: | Judgment: | Judgment: |  |
|  | Support:  A random sequence allocation was performed using block randomization | Support:  Allocation was concealed in sealed, opaque envelopes. | Support:  Neither trial personnel nor participants were blinded due to the nature of the study; however, no deviations from the intended intervention were observed. | Support:  Approximately 36 participants (12.6%) were lost to follow-up, with 16 from the intervention group and 20 from the control group. The reasons for dropout were described. | Support:  There was no report on how Hb measurements were taken, and outcome assessors were aware of the intervention. | Support:  The authors reported the expected outcomes  per the pre-specified plan. Clinical Trial Registry Number available. | **Some**  **Concerns** |
| Abujilban et al [45] | Judgment: | Judgment: | Judgment: | Judgment: | Judgment: | Judgment: |  |
|  | Support:  A random sequence allocation was performed using a computer-generated table of random numbers. | Support:  The method used for allocation sequence concealment was not reported | Support:  There was no report on blinding or how deviations from the intended intervention were minimized. | Support:  Approximately 8 participants (3.8%) were lost to follow-up, with 5 from the intervention group and 3 from the control group. The reasons for the lost to follow-up were described. | Support:  There was no report on how Hb measurements were taken, and it is unclear whether outcome assessors were blinded. | Support:  The authors reported the study outcomes, but it is unclear whether they align with the pre-specified plan. Neither study protocol nor proof of Clinical Trail Registry is available. | **Some**  **Concerns** |

|  | Low risk of bias |
| --- | --- |
|  | Some concerns |

Domain 1: Bias arising from the Randomization process.

Domain 2: Bias due to deviations from intended interventions.

Domain 3: Bias due to missing outcome data.

Domain 4: Bias in measurement of the outcome data.

Domain 5: Bias in selection of the reported results.

Summary of the risk of bias assessment


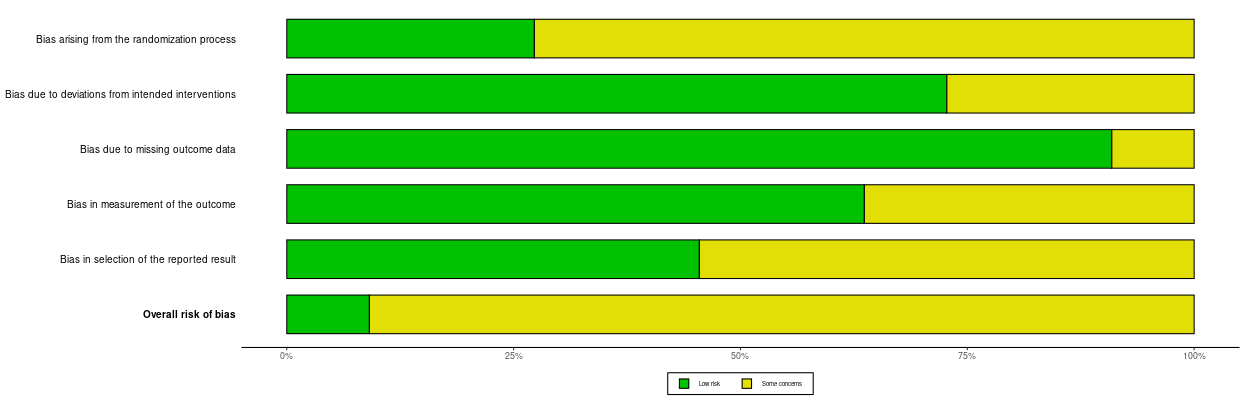

Supplement: Multimedia Appendix 3 [file mhealth-v14-e81001-s003.docx]
